# Human Breast Milk NMR Metabolomic Profile across Specific Geographical Locations and Its Association with the Milk Microbiota

**DOI:** 10.3390/nu10101355

**Published:** 2018-09-21

**Authors:** Carlos Gómez-Gallego, Jose Manuel Morales, Daniel Monleón, Elloise du Toit, Himanshu Kumar, Kaisa M. Linderborg, Yumei Zhang, Baoru Yang, Erika Isolauri, Seppo Salminen, Maria Carmen Collado

**Affiliations:** 1 Functional Foods Forum, Faculty of Medicine, University of Turku, 20014 Turku, Finland; kumar.himanshu@utu.fi (H.K.); sepsal@utu.fi (S.S.); 2 Laboratory of Metabolomics, Institute of Health Research-INCLIVA, 46010 Valencia, Spain; J.Manuel.Morales@uv.es (J.M.M.); daniel.monleon@uv.es (D.M.); 3 Unidad Central de Investigación en Medicina, University of Valencia, 46010 Valencia, Spain; 4 Pathology Department, School of Medicine, University of Valencia, 46100 Valencia, Spain; 5 Division of Medical Microbiology, Department of Pathology, University of Cape Town, 7925 Cape Town, South Africa; elloisedutoit@gmail.com; 6 Food Chemistry and Food Development, Department of Biochemistry, University of Turku, 20014 Turku, Finland; kamayl@utu.fi (K.M.L.); bayang@utu.fi (B.Y.); 7 Department of Nutrition and Food Hygiene, School of Public Health, Peking University, 100191 Beijing, China; zhangyumei@bjmu.edu.cn; 8 Department of Paediatrics, University of Turku and Turku University Hospital, 20520 Turku, Finland; eriiso@utu.fi; 9 Department of Biotechnology, Institute of Agrochemistry and Food Technology-National Research Council (IATA-CSIC), 46980 Valencia, Spain

**Keywords:** human milk, metabolites, microbiome, mode of delivery, caesarean section, proton nuclear magnetic resonance

## Abstract

The composition of human breast milk is highly variable, and it can be influenced by genetics, diet, lifestyle, and other environmental factors. This study aimed to investigate the impact of geographical location and mode of delivery on the nuclear magnetic resonance spectroscopy (NMR) metabolic profile of breast milk and its relationship with the milk microbiome. Human milk metabolic and microbiota profiles were determined using NMR and 16S rRNA gene sequencing, respectively, in 79 healthy women from Finland, Spain, South Africa, and China. Up to 68 metabolites, including amino acids, oligosaccharides, and fatty acid-associated metabolites, were identified in the milk NMR spectra. The metabolite profiles showed significant differences between geographical locations, with significant differences (*p* < 0.05) in the levels of galactose, lacto-*N*-fucopentaose III, lacto-*N*-fucopentaose I and 2-fucosyllactose, 3-fucosyllactose, lacto-*N*-difucohexaose II, lacto-*N*-fucopentaose III, 2-hydroxybutyrate, 3-hydroxybutyrate, proline, *N*-acetyl lysine, methyl-histidine, dimethylamine, kynurenine, urea, creatine and creatine phosphate, formate, lactate, acetate, phosphocholine, acetylcholine, LDL, VLDL, ethanolamine, riboflavin, hippurate, spermidine, spermine and uridine. Additionally, the effect of caesarean section on milk metabolome was dependent on the geographical region. Specific interrelations between human milk metabolites and microbiota were also identified. Proteobacteria, Actinobacteria, and Bacilli were most significantly associated with the milk metabolites, being either positively or negatively correlated depending on the metabolite. Our results reveal specific milk metabolomic profiles across geographical locations and also highlight the potential interactions between human milk’s metabolites and microbes.

## 1. Introduction

Arguably, the cornerstone of healthy growth and development in children is breast feeding, since breast milk offers myriad physiological advantages when compared to other sources of nutrition. Indeed, the optimal model of infant feeding is the healthy breastfed child [1]. When compared with formula-fed infants, breastfed children exhibit a reduced risk of gastrointestinal and respiratory infections [2], allergic disease [3,4], and being overweight or obese, with the benefits actually extending beyond infancy [5,6].

Breast milk contains many biologically active compounds, such as growth factors, antimicrobial and immune-enhancing substances, oligosaccharides (HMOs), as well as a diverse and rich bacterial community [7,8,9,10].

The composition of human breast milk is determined by genetic factors, lifestyle, diet, and the age of the mother [11,12]. Furthermore, human milk’s macronutrient composition varies across lactation, although it is relatively conserved between populations [13]. However, the determinants of the bioactive compounds are currently less well understood. While some components of human milk appear to be relatively stable across different locations, some, such as polyunsaturated fatty acids, vary according to the mother’s diet [9,14], while others, such as polyamines [15], HMOs [16], and milk bacteria [9,17], seem to vary depending of multiple factors. The mode of birth has also been reported to be a modulating factor for human milk’s composition, acting in a differential manner in different countries [9,15]. Yet, the complex interactions between the constituents of milk, the biological impact, and the consequences for the infant’s health in the short- and long-term remain unclear.

In addition, the host-milk microbial interactions may be influenced by the presence and/or concentration of milk metabolites, which may in turn influence the intestinal bacterial communities as well as the immune cell populations in breastfed children due to favoring the growth of specific microbial genera [8].

The present study aimed to compare the metabolomic profile of human milk obtained from different regions and different delivery modes, as well as to ascertain the potential interaction with milk microbiota.

## 2. Materials and Methods

### 2.1. Breast Milk Sample Collection

This study’s population comprised 79 healthy women volunteers representing different populations from around the world, including China (Beijing area), South Africa (Cape Town area), Finland (southwestern area), and Spain (Valencia area). The women were enrolled in the study according to previously described inclusion criteria [9]. Further, the subjects from each country (n = 20) were grouped into two sub-groups according to the mode of delivery, namely either vaginal delivery (n = 10 for each country) or caesarean section (n = 10 for China, Finland, and Spain; n = 9 for South Africa). Data regarding age of the mother and perinatal body max index (BMI) are presented in Table S1. The mothers from China had significantly lower BMI (*p* < 0.001), but there were no differences in other parameters between countries and mode of delivery. Parity was between 1 and 3, mostly 1 and 2, with no differences among countries or between mode of delivery. Exclusively breastfeeding was reported at time of the sampling.

All the participating women received written, complete, and detailed information about the study. Written informed consent was obtained from all the participants, and the ethics committees of the respective participating countries (Spain [Bioethics Committee of CSIC and the Regional Ethics Committee for Biomedical Research, Ref: ERC-639226], Finland [Turku University Hospital, Ref: 24/1801/2013], China [Medical Research Board of Peking University, Ref: IRB00001052-16038], and South Africa [University of Cape Town, Human Research Ethics Committee, Ref: HREC 649/2016]), approved the study protocol. The study was conducted in accordance with the Declaration of Helsinki. Inclusion criteria requested exclusive breastfeeding practices at sampling time and healthy status of the mother-child pairs. Exclusion criteria included antibiotics use after birth, perinatal probiotic consumption, and presence of disease.

Prior to the sample collection, the mothers were given oral and written instructions regarding the standardized collection of samples. The mature milk samples (one month postpartum) were collected manually in the morning into a sterile tube using the same protocol in all the countries. Before the collection, the mothers’ nipples and mammary areola were cleaned with soap and sterile water and then soaked in chlorohexidine in order to reduce the presence of skin bacteria. Samples were collected in the morning, from one breast, before baby feeding. The first drops of milk (approx. 500 μL) were discarded. The average collected volume was 10 mL. All the samples were kept frozen at −20 °C until delivery to the laboratory. They were then stored at −80 °C for further analysis.

The breast milk samples were thawed, carefully mixed by means of inversion, and then centrifuged at 14,000 rpm for 20 min at 4 °C. The fat was removed and the pellet was used for the total DNA extraction. Avoiding the outer layer of fat, the whey milk was transferred to a clean Falcon tube and then centrifuged again. This procedure was repeated. A clear supernatant was used for the metabolomic profile analysis.

### 2.2. Breast Milk Metabolite Profiling

A proton nuclear magnetic resonance (NMR) analysis of all the collected samples was performed. For each group, the milk samples (455 µL) were mixed with 45 µL of sodium-3′-trimethylsilylpropionate-2,2,3,3-d4 (TSP) solved in deuterium oxide and then placed in a 5 mm NMR tube. The final TSP concentration in each sample was 2.5 mM. All the spectra were recorded on a Bruker Avance DRX 600 spectrometer (Bruker GmbH, Rheinstetten, Germany) operating at a ^1^H frequency of 600.13 MHz. The spectrometer was equipped with a triple resonance ^1^H/^13^C/^31^P probe. The nominal temperature of the samples was kept at 310 K. A single-pulse pre-saturation experiment was performed for all the samples. A total of 64 transients were collected into 65 k data points for all the experiments, with a spectral width of 14 ppm. Water presaturation was performed for one second during the recycling delay for the solvent signal suppression. Prior to the Fourier transformation, the free induction decay was multiplied with a 0.3 Hz exponential line-broadening function. All the spectra were processed using MestReNova 8.1 software (Mestrelab Research S.L., Santiago de Compostela, Spain) and then transferred to MATLAB R2013a (The MathWorks Inc., Natick, MA 2013) using in-house scripts for data analysis. The metabolite spin systems and resonances were identified using data obtained from both the literature and the commercial resonances database Chenomx NMR Suite Profiler (Chenomx NMR Suite 8.1, Chenomx Inc., Edmonton, AB, Canada). The spectra were manually phase corrected and baseline adjusted, referenced to the TSP, and normalized to the total aliphatic spectral area (0.50 and 4.40 ppm) in order to eliminate any differences in the total metabolite concentration. The signals belonging to the identified metabolites were then integrated and quantified using the semi-automated ^1^H NMR signal deconvolution routines in MestReNova 8.1 (Mestrelab Research SL, Santiago de Compostela, Spain). The final metabolite levels were calculated in arbitrary units as the area under the peak. In addition, two-dimensional NMR methods, including homonuclear correlation spectroscopy (TOCSY) and heteronuclear single quantum correlation spectroscopy (HSQC), were applied to a selected group of samples so as to confirm the assessment of the metabolites.

### 2.3. Breast Milk DNA Extraction and Microbial 16S rRNA Gene Sequencing

The process of microbial DNA extraction and sequencing using an Illumina MiSeq sequencer was described in a previous study by Kumar et al. [9]. The sequencing data were submitted to the National Center for Biotechnology Information with the Sequence Read Archive accession: SRP082263 and submission ID: SUB1772296.

### 2.4. Statistical Analysis

A chemometrics statistical analysis was performed using in-house MATLAB scripts and the PLS_Toolbox 8.0.2 (Eigenvector Research, Inc., Wenatchee, WA, USA) statistical multivariate analysis library. The normalization of the NMR spectra was done using the total aliphatic spectral area (0.50 and 4.40 ppm) in order to eliminate any differences in the total metabolite concentration. Mean-centered and Pareto data scaling were used prior to multivariate analysis. A principal component analysis (PCA) was applied to the NMR spectra data sets. A PCA is able to identify low-dimensional embeddings of multivariate data in such a way that optimally preserves the structure of the data. The main advantage of PCA models is that the key sources of variability within the data are modeled by the so-called principal components (PCs) and, consequently, their associated scores and loadings allow for the visualization and understanding of different patterns and relations in the data. The principal components were chosen to explain at least 70% of the variance. The loading plots of the corresponding principal components were then used to detect the positions of most discriminative variables in the NMR spectra. In order to maximize the separation between the samples, a partial least squares discriminant analysis (PLS-DA) was conducted. A permutation test was performed to check the overfitting of the PLS-DA models. The multivariate chemometric models were cross-validated using ten-fold leave-one-out cross-validation. In each run, 10% of the data were left out of the training and used to test the model. The entire cross-validation process was run ten times. The spectral regions responsible for the classification of the models were identified using the variable importance in projections (VIP) coefficients obtained during the PLS-DA. The threshold used for VIP selection was ≥1. Spectral regions with high VIP coefficients are more important in terms of providing class separation during the analysis, while those with very small VIP coefficients provide only a small contribution to the classification.

SPSS 25.0.0.1 software (IBM Corp., Armonk, NY, USA) was employed for the statistical analysis of the milk metabolites. Differences were considered significant at *p* ≤ 0.05. Due to the non-normal distribution of the data and high presence of outliers, nonparametric tests were used. Comparisons among the data between the different countries were made by applying the Kruskal-Wallis test, while comparisons between the modes of birth were made by applying the Mann-Whitney U test. The significance values in the pairwise comparison were adjusted using the Bonferroni correction for multiple tests.

Calypso online software version 8.50 was used for data normalized via cumulative sum scaling in order to generate heat maps for the Spearman’s correlations between the microbial groups and milk metabolites.

## 3. Results

A total of 68 metabolites were identified in the human milk one month after delivery, as detailed in Table 1. The metabolites included 23 amino acids and derivatives, 18 sugars and derivatives, ten lipids and fatty acid-associated metabolites, and seven metabolites associated with energy metabolism, while the rest were linked to metabolic processes involving vitamins or nucleic acids, microbial metabolism, and food additives.

The most abundant metabolite was lactose, followed by lipids, with high amounts of the lipoproteins LDL (low-density lipoprotein) and VLDL (very low-density lipoproteins), and then, HMOs, and amino acids. The accurate relative quantitation of several metabolites by means of NMR was difficult due to the presence of multiple peaks or severe spectral overlapping, and it was thus not included in statistical analysis. Therefore, of the 68 metabolites identified, 37 were employed for statistical analysis in SPSS 25.0.0.1 software. Maternal factor and metabolites association were analyzed as shown in Table S2.

The presence of LDL and VLDL as milk metabolites can be controversial. The particles detected in milk have similar nuclear magnetic resonances (NMR), physicochemical properties and mobility than those lipoproteins detected in plasma. However, they might be also different lipid and protein conjugates with similar composition and hydrodynamic properties; lipids similar to those present in LDL and VLDL enclosed in phospholipids, free cholesterol and proteins; or free lipids similar to those enclosed in LDL and VLDL attached to large proteins. For this reason, they should be considered LDL- and VLDL-like particles.

### 3.1. Differences in Milk Metabolites between Countries

**Figure 1:**
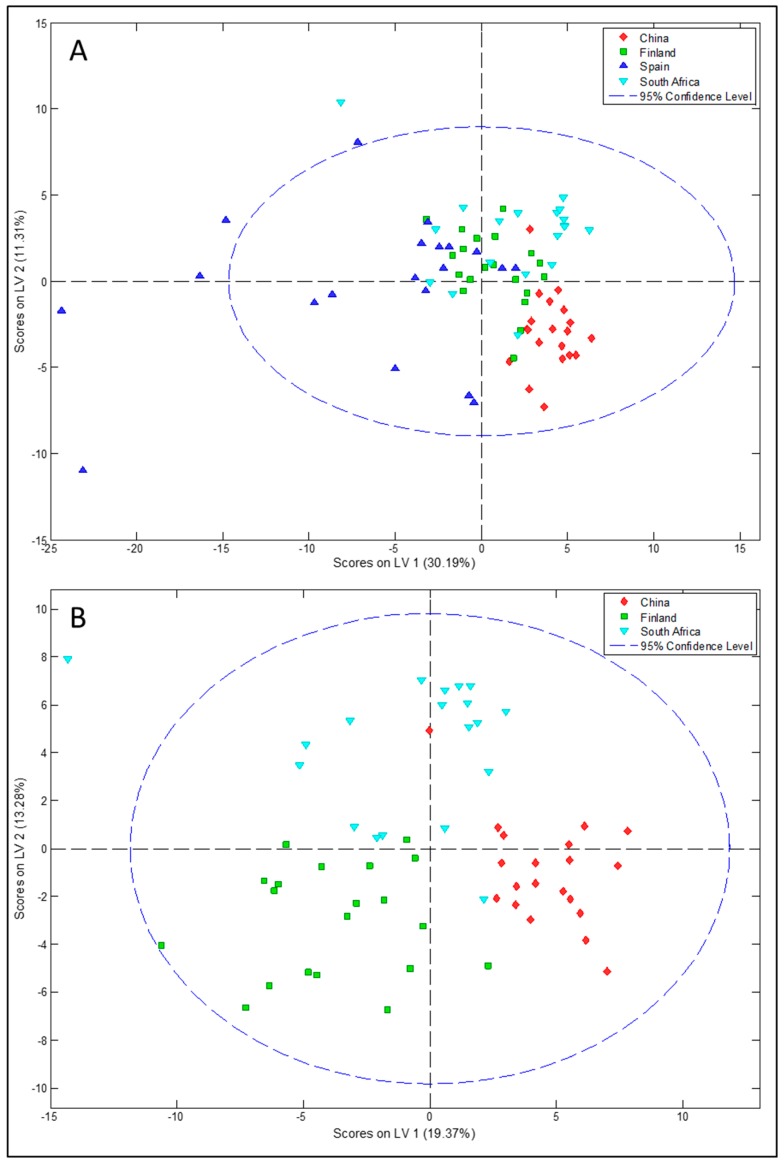
Partial least squares discriminant analysis (PLSDA) scores plot scaling nuclear magnetic resonance (NMR) data from all participant countries (**A**); participant countries without Spain (**B**). Countries are indicated as red diamonds (China), green squares (Finland), navy blue triangles (Spain), pale blue triangles (South Africa).

The global metabolic profile of breast milk was found to be different between countries, as seen in Figure 1. The PLS-DA analysis showed the Spanish samples to be widely dispersed, being more similar to the Finnish and South African samples and totally separate from the Chinese samples. After the exclusion of the Spanish milk samples from the analysis, total separation with only minimal overlapping between the samples was observed. PCA plots and loadings are available as supplementary material in Figures S1 and S2.

**Figure 2:**
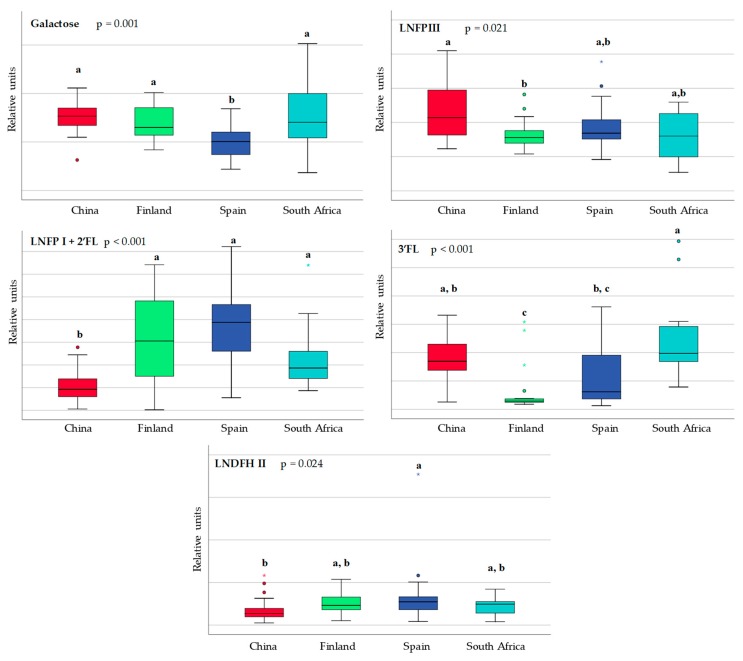
Box and whisker plot showing statistical significant differences in sugars and derivatives in breast milk obtained from China (red), Finland (green), Spain (blue) and South Africa (pale blue). Each bar represents the smallest observation, lower quartile (**Q1**), median, upper quartile (**Q3**) and largest observation. Differences among countries were calculated using the Kruskal-Wallis test. Unlike letters indicate statistically significant differences among countries. Circles and stars indicate outlier data. 3′FL: 3-fucosyllactose; LNFP I + 2′FL: lacto-*N*-fucopentaose I and 2-fucosyllactose; LNDFHII: lacto-*N*-difucohexaose II; LNFP III: lacto-*N*-fucopentaose III. Data are expressed in relative units.

Significant differences in the sugars and HMOs between the countries can be seen in Figure 2. When compared to the breast milk samples from Finland, the Chinese samples exhibited significantly higher levels of 3-fucosyllactose (3′FL) and lacto-*N*-fucopentaose III (LNFP III). A higher abundance of lacto-*N*-fucopentaose I (LNFP I) and 2-fucosyllactose (2′FL) was observed in Finland and Spain, respectively, while 3′FL and LNFP III were more highly abundant in South Africa and China, respectively.

**Figure 3:**
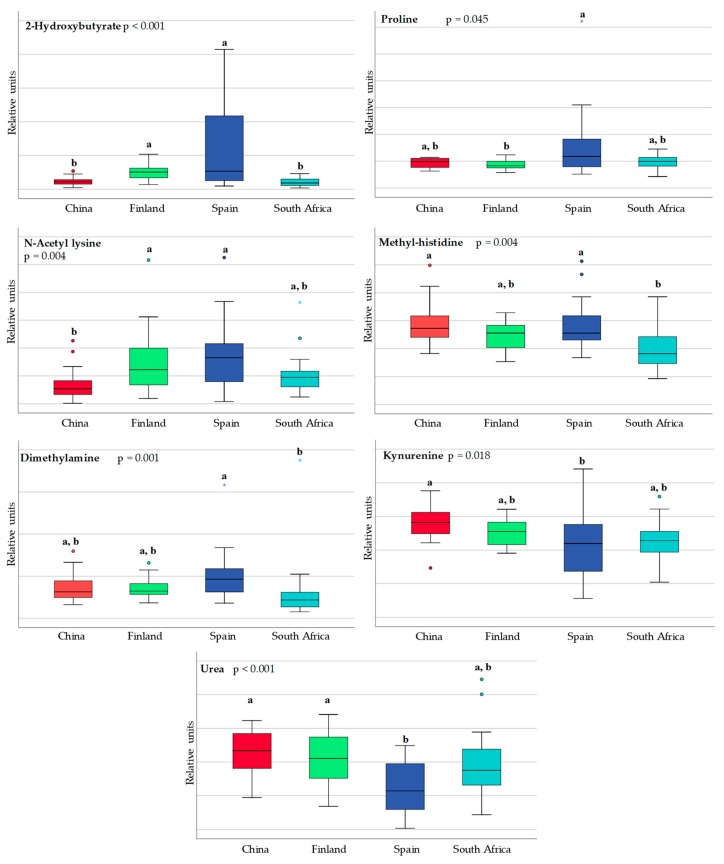
Box and whisker plot showing statistical significant differences in amino acids and derivatives in breast milk obtained from China (red), Finland (green), Spain (blue) and South Africa (pale blue). Data are expressed in relative units. Each bar represents the smallest observation, lower quartile (**Q1**), median, upper quartile (**Q3**) and largest observation. Differences among countries were calculated using the Kruskal-Wallis test. Unlike letters indicate statistically significant differences among countries. Circles and stars indicate outlier data.

Figure 3 shows statistically significant regional differences in the amino acids and derivatives found in the human milk samples. All of them have an endogenous origin and might therefore be the result of dietary and/or metabolic differences between geographical locations.

**Figure 4:**
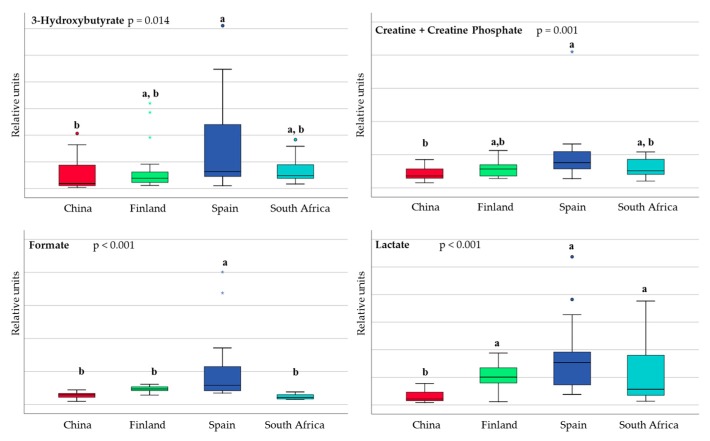
Box and whisker plot showing statistical significant differences in energy metabolites in breast milk in samples from China (red), Finland (green), Spain (blue) and South Africa (pale blue). Data are expressed in relative units. Each bar represents the smallest observation, lower quartile (**Q1**), median, upper quartile (**Q3**) and largest observation. Circles and stars indicate outlier data. Differences among countries were calculated using the Kruskal–Wallis test. Unlike letters indicate statistically significant differences among countries.

**Figure 5:**
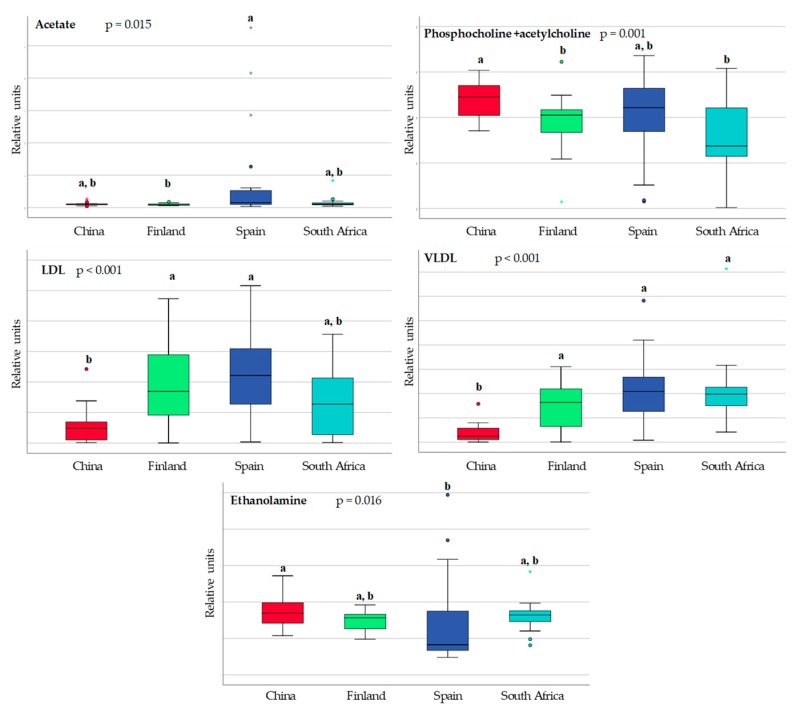
Box and whisker plot showing statistical significant differences in fatty acids and related metabolites in breast milk in samples from China (red), Finland (green), Spain (blue) and South Africa (pale blue). Data are expressed in relative units. Each bar represents the smallest observation, lower quartile (**Q1**), median, upper quartile (**Q3**) and largest observation. Circles and stars indicate outlier data. Differences among countries were calculated using the Kruskal–Wallis test. Unlike letters indicate statistically significant differences among countries. LDL and VLDL should be consider LDL- and VLDL-like particles with similar nuclear magnetic resonances (NMR), structure and mobility than plasma LDL and VLDL.

With regard to the content of the energy metabolites, fatty acids, and associated metabolites, the differences between countries are shown in Figure 4 and Figure 5. The Finnish and Spanish samples were characterized by higher levels of lipoproteins (LDL and VLDL). Short-chain fatty acids (SCFA) were also detected with higher levels of acetate and formate in the Spanish samples.

**Figure 6:**
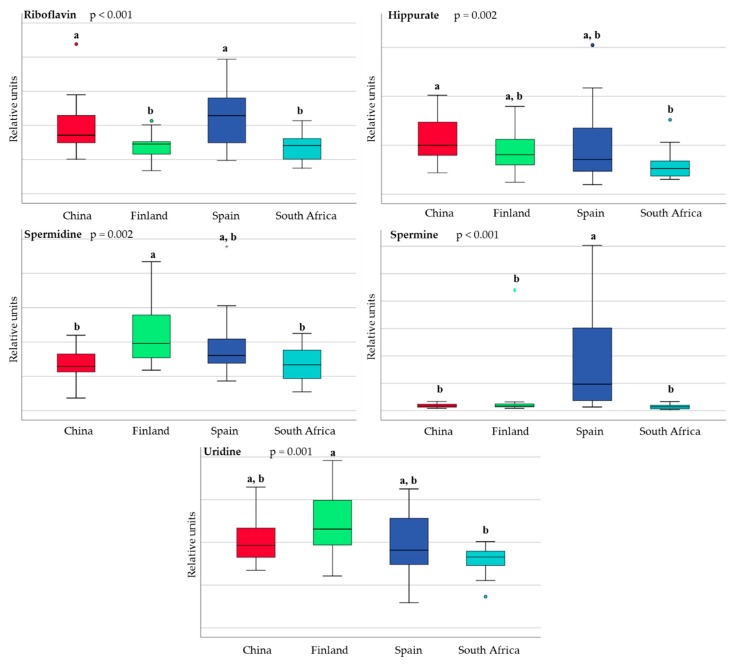
Box and whisker plot showing statistical significant differences in other metabolites in breast milk in samples from China (red), Finland (green), Spain (blue) and South Africa (pale bue). Data are expressed in relative units. Each bar represents the smallest observation, lower quartile (**Q1**), median, upper quartile (**Q3**) and largest observation. Circles and stars indicate outlier data. Differences among countries were calculated using the Kruskal–Wallis test. Unlike letters indicate statistically significant differences among countries.

The statistically significant differences identified in the other metabolites are presented in Figure 6. Some of them have a dietary origin, such as vitamin B2 (riboflavin), while others, such as the polyamines (spermidine and spermine), could have an endogenous, dietary, or microbial origin.

### 3.2. Impact of Mode of Delivery on Human Milk Metabolites

**Figure 7:**
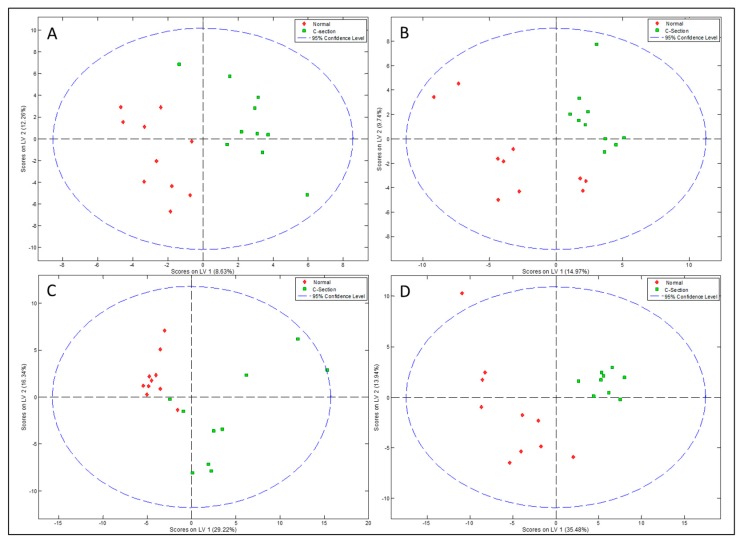
Partial least squares discriminant analysis scores plot scaling nuclear magnetic resonance (NMR) data from vaginal delivery samples (red) and caesarean section (green) from China (**A**), Finland (**B**), Spain (**C**), South Africa (**D**).

Independent of the country, the mode of delivery has a distinct impact on the human milk metabolome, as seen in Figure 7.

When considering all the mothers included in the study, six of the 37 semi-quantified metabolites were statistically different in the milk samples obtained from the mothers who underwent vaginal delivery when compared to those who underwent caesarean section, as seen in Figure S3. The milk from the mothers who underwent vaginal delivery had higher levels of 3-hydroxybutyrate (*p* = 0.048) and LNFP III (*p* = 0.045), while the milk from the mothers who underwent caesarean section had higher relative abundances of butyrate (*p* = 0.043), ethanolamine (*p* = 0.004), proline (*p* = 0.018), and urea (*p* = 0.020). However, these differences depend on the country. In the present study, based on the mode of delivery, we found significant differences in 16 metabolites in the South African samples, 11 metabolites in the Spanish samples, four in the Finnish samples, and one in the Chinese samples, shown in Table 2, reflecting regional differences in terms of the impact of caesarean section on the milk metabolome.

### 3.3. Relationship between the NMR Metabolomic Profile and Milk Microbiota

**Figure 8:**
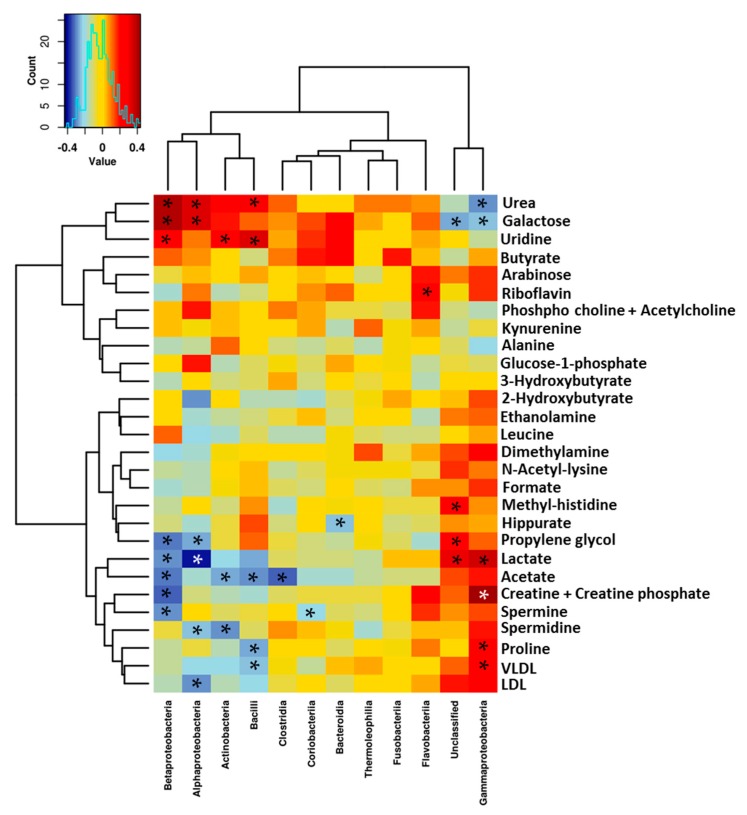
Heat map to show Spearman’s correlation between metabolites and microbiota composition at class level. Asterisk indicates statistically significant correlation at the level of (*p* < 0.05). LDL and VLDL should be considered LDL- and VLDL-like particles with similar nuclear magnetic resonances (NMR), structure and mobility than plasma LDL and VLDL.

**Figure 9:**
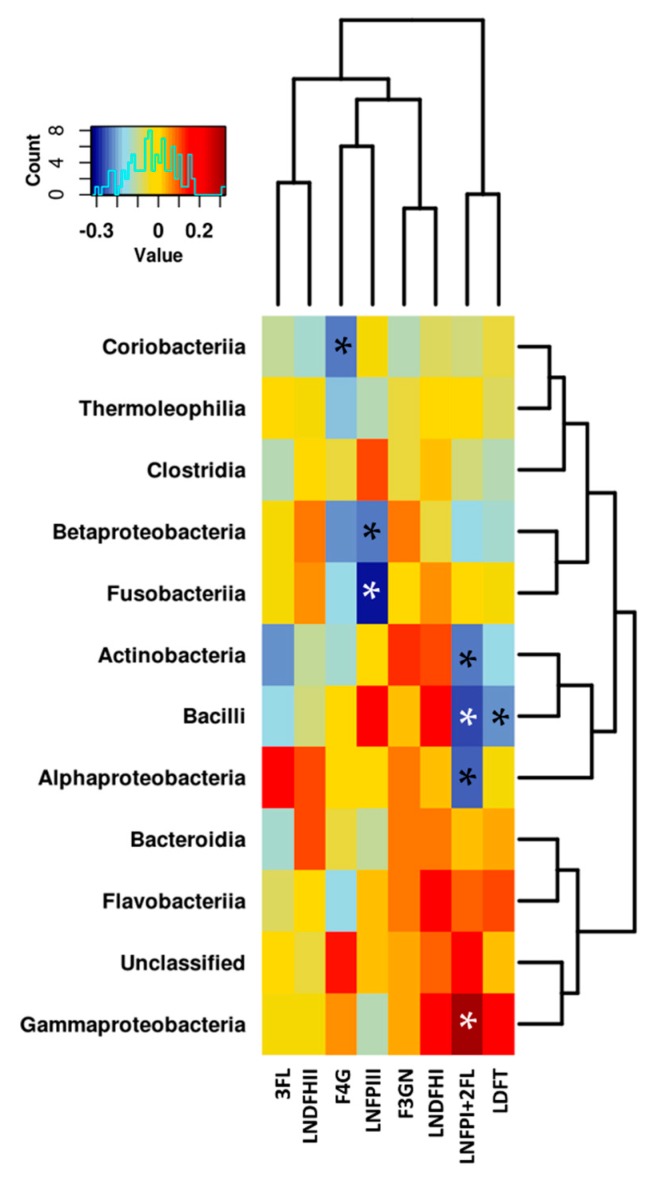
Heat map to show Spearman’s correlation between oligosaccharides (HMOs) and microbiota composition at class level. 3FL: 3-fucosyllactose; LNDFHII: Lacto-*N*-difucohexaose; F4G: Fucosyl-α-1,4-*N*-acetylglucosamine; LNFP III: lacto-*N*-fucopentaose; F3GN: Fucosyl-α-1,3-*N*-acetylglucosamine; LNDFH I: lacto-*N*-difucohexaose I; LNFP I: lacto-*N*-fucopentaose I; 2FL: 2-fucosyllactose; LDFT: lactodifucotetraose. Asterisk indicates statistically significant correlation at the level of (*p* < 0.05).

In order to explore the interrelations between the human milk metabolites and the milk microbiota profile, Spearman’s rank correlations were determined and then represented in heat maps shown in Figure 8 and Figure 9. Urea and galactose were positively correlated (*p* < 0.05) with Alpha- and Betaproteobacteria and Bacilli, although they were negatively correlated (*p* < 0.05) with Gammaproteobacteria. Yet, a group of metabolites were found to be positively correlated (*p* < 0.05) with Gammaproteobacteria, including lactate, creatine, proline, lacto-*N*-fucopentaose I, and 2-fucosyllactose VLDL, although they negatively correlated (*p* < 0.05) with Alpha- and Betaproteobacteria and Bacilli.

The Actinobacteria in the human breast milk were positively correlated (*p* < 0.05) with uridine, but negatively correlated with lacto-*N*-fucopentaose I, 2-fucosyllactose acetate, and spermidine (*p* < 0.05) as seen in Figure 8.

A few significant associations were found between the bacterial groups and HMOs, namely those related to fucosyl-α-1,4-*N*-acetylglucosamine, lactodifucotetraose, lacto-*N*-fucopentaose III, lacto-*N*-fucopentaose I, and 2-fucosyllactose, shown in Figure 9.

## 4. Discussion

There is growing research interest in identifying and understanding the bioactive compounds found in human breast milk. The field of infant nutrition research is evolving and new methodologies are being developed to support exact knowledge concerning the composition of human milk. Moreover, a few prior studies have reported the composition of human milk using NMR-based metabolomics [1,7,19,20]. In this study, a total of 68 metabolites were identified in mature human milk. To the best of our knowledge, this is the first study to report the distinct milk metabolomics profile across different geographical locations, as well as to associate the complex interactions with human milk microbes. Spanish samples presented higher dispersion that was no associated with differences in maternal characteristics such as parity, age, or BMI between samples. Future studies to find the origin of this variability will be needed.

Different mode of delivery (vaginal vs caesarean section) influences the milk metabolite profile across locations. This study hence shows for the first time regional differences in the impact of caesarean section on human milk metabolites. The effect of caesarean section on the milk metabolome is dependent on the geographical region, with changes in different metabolites depending on the country. We suggest that the clinical intervention during caesarean and the alterations in the physiological and hormonal signals produced during normal vaginal delivery, would affected the milk metabolite profiles as already showed for milk microbiota [9]. In addition, we observed that the impact of the mode of delivery vary among geographical locations, maybe due to differences in the clinical procedures and antibiotic use [9].

Previous studies have shown inter-individual variations in human milk in terms of the macronutrients [21]. With regard to the metabolites, some of them, such as lactose, *myo*-inositol, and urea, have been reported to be conserved among mothers due to their important roles in infant growth and development, while those metabolites related to the genetic background or maternal diet are more variable, including HMOs, amino acids, choline, and vitamins [1,20]. No significant changes were found in the lactose and *myo*-inositol levels in relation to the geographical location and mode of delivery in this study, which suggests the strict regulation by the mammary gland, as has been proposed previously [1]. Yet, previous studies have found significant differences with regard to urea. For instance, Smilowitz et al. [1] analyzed 52 human milk samples collected at day 90 postpartum. They found that urea was one of the most abundant metabolites in human milk, and they further reported low variability between mothers, which suggested regulation at the level of the mammary gland, as well as an important role in developing infants as a source of nitrogen for intestinal microbiota. In the present study, the samples collected at day 30 postpartum showed differences in urea at different geographical locations in addition to relatively high variability. This may indicate differences in the regulation of the mammary gland depending on the number of days postpartum, as well as the influence of external factors, such as meat and dairy intake, or physical activity, during the early stages of lactation. The differences in the urea composition across the different geographical locations could also reflect variation in the gut microbiota composition of infants from the different locations [9,22,23]. A positive correlation between the colonic urea-nitrogen metabolism and the bifidobacteria concentration has previously been reported [24], although this association between the nitrogenated compounds and bifidobacteria might start early. We identified positive correlation between the nitrogenated compounds, such as urea, and uridine and Actinobacteria.

Similarly to urea, riboflavin has previously been reported to be a stable milk constituent in well-nourished mothers during the first month of lactation [19], although in the present study, its level varied with geographical locations. These differences might be explained by differences in maternal diet [20], and it might be extrapolated to other milk metabolites.

When compared with the other constituents, fat has previously been classified as the most variable component of breast milk due to being influenced by the time of day, inter-feeding interval, point of sampling during a feeding, stage of lactation, maternal weight, and differences between breasts [25]. In the present study, the differences in the LDL and VLDL in the milk might be partially explained by dietary impacts [26] or genetic factors, as previously reported [27]. However, their role in infant nutrition remains unclear, and the fatty acid content and their composition inside the lipoproteins might be more important for the infant than the content of lipoprotein particles. A recent study has shown the difference in the lipidomic profile across countries [9]. In this study, the lipid composition, particularly that of polyunsaturated fatty acids (PUFA), differed between the countries, with the highest level being observed in the case of omega-6 PUFA in Chinese women.

Human milk oligosaccharides, which are the third most abundant component in human milk [28,29], are a group of complex sugars that are non-digestible by infants. These HMOs contain a lactose core bound to one or more glucose, galactose, *N*-acetylglucosamine, fucose, or sialic acid residues [30]. HMOs support the competitive growth of beneficial bacterial strains within the intestine [29], inhibit the adhesion of pathogens to the infant’s epithelium, and interact directly with host immune cells [30]. Each HMO fulfils different roles and activities, and each is metabolized by different bacteria [31]. In addition, specific HMOs could render epithelial cells more resistant to bacterial colonization, and they can interact with immune cells in order to reduce the expression of pro-inflammatory cytokines [32]. This means that differences in the HMOs between geographical locations may result in different roles for human milk in relation to infant health and development. It has been suggested that HMO concentrations and profiles may vary geographically [16,32]. The present study confirmed significant differences in both sugars and HMOs between countries, which was also revealed by a study by McGuire et al. [16]. Their study revealed that milk from Sweden contained more than four times more 3-fucosyllactose (3FL) and lower disialyllacto-*N*-tetraose than milk collected in rural Gambia. Similarly, our study showed significant differences in the 3FL, lacto-*N*-fucopentaose III (LNFPIII), lacto-*N*-fucopentaose I (LNFPI), and 2-fucosyllactose (2FL) levels. The HMO profile and specific oligosaccharides, such as 2FL, have also been linked to infant body composition and growth [33], the survival of children born to HIV-infected mothers [34], and allergic morbidity such as a cow milk protein allergy [33]. Furthermore, it was previously reported that specific HMOs, such as LNFPI and 3′-sialyllactose, were associated with infant morbidity and growth development, respectively, and at the same time, correlated with specific microbiota [31]. Another prior study reported an association between the total HMO concentrations and Actinobacteria (mainly *Bifidobacterium* spp.) counts [35]. More specifically, it was reported that *B. breve* was positively correlated with sialylated HMOs, while *B. longum* was positively correlated with non-fucosylated/non-sialylated HMOs. Furthermore, the same study reported positive correlations between fucosylated HMOs and the classes Verrucomicrobiae (*Akkermansia muciniphila*) and Bacilli, mainly *Staphylococcus aureus* [35]. Our results thus support the available, albeit limited, data concerning the complex interactions between microbiota and HMOs during lactation, with significant associations being found between the bacterial groups and fucosyl-α-1,4-*N*-acetylglucosamine, lactodifucotetraose, LNFPIII, LNFPI, and 2FL. According to this study, geographical variations and changes in the metabolomics profile of human milk following caesarean section may involve differences in the protective activities of HMOs.

Previous studies have indicated that the neonatal primary gut colonizers include acetate- and lactate-producing bacteria from the classes Actinobacteria (*Bifidobacterium*), Bacteroidia (*Bacteroides*) and Bacilli (*Lactobacillus*, *Streptococcus*, *Staphylococcus*, and *Enterococcus*) [36]. These bacteria have also been found to be present in human breast milk in its viable form [37,38]. In this study, higher amounts of lactate, followed by acetate and propionate, were detected in the human milk samples across locations. The origin of this lactate may be both the mammary tissue and/or milk microbiota. In the mammary gland, lactate serves as an intermediate in carbohydrates metabolism [39], while lactate and acetate are intermediate fermentation products of the microbial metabolism [40,41]. The present study found significant differences in the acetate and lactate levels between locations, which were also correlated with several bacterial groups. The impact of milk lactate on infant health is not yet known, although recent studies have suggested that lactate must be efficiently metabolized during early life in order to avoid the potential negative consequences of lactate accumulation as acidosis, neurotoxicity, and cardiac arrhythmia [42]. In addition, it is known that acetate is a product of *Bifidobacterium* metabolism, and it serves to promote the defense functions of the host cells and exert a protective effect against infection [43]. It has also been established that acetate and lactate produced by *Bifidobacterium* and *Lactobacillus* contribute to SCFA-mediated health effects, although these two microorganisms do not directly produce butyrate and/or propionate [43,44].

Interestingly, ethanol was also identified in the breast milk samples analyzed in our study. Considering that mothers do not generally drink alcoholic beverages during lactation, the presence of this compound in small amounts in human milk might be a consequence of milk microbiota metabolism. Many microorganisms, including bacteria and yeasts, produce ethanol as the major fermentation product of carbohydrates. Furthermore, recent studies have reported the presence of yeasts, such as *Saccharomyces* and *Candida*, in breast milk samples obtained from healthy women [45], and the presence of ethanol in milk might be a product of their metabolism.

It has previously been reported that specific interactions take place between milk microbes and other milk components, including macro- and micro-nutrients as well as milk cells [8,10]. In this study, we found a group of metabolites to be positively correlated with Gammaproteobacteria, including lactate, creatine, proline, lacto-*N*-fucopentaose I, and 2-fucosyllactose VLDL, as well as negatively correlated with Alpha- and Betaproteobacteria, and Bacilli.

The major limitation of this study is that we were unable to determine the effect of geographical location and caesarean section alone on the milk metabolome without the influence of other confounding variables (e.g., gestational age, diet, lifestyle, secretor status). To do so, future studies involving higher sample sizes and controlling for such variables should be performed. Despite this limitation, the present study contributes important information regarding regional variations in human milk metabolites, the impact of caesarean section, and the correlation of milk metabolites with milk microbiota. Understanding both the composition and function of the components of milk is vital to infant health. The data obtained from this study could form the basis for future studies concerning the milk metabolome and their contribution to infant health and development.

Taken together, ethnicity, diet, environment, and lifestyle could partially explain the different NMR metabolomic profiles seen in this study. It is well-known that diet influences the human metabolomics profile in the blood [46], urine [47], tissues [47], and fecal supernatants [48], and something similar may be expected in human milk. Moreover, only a few studies have investigated the relationships among the human milk microbiota and milk constituents [12,14]. Our findings confirm prior evidence showing that complex bacterial communities within milk are associated with variations in the nutritional composition and metabolites profiles [9,15].

## 5. Conclusions

By means of NMR, we detected 68 metabolites in human milk sampled one month postpartum. The concentrations of carbohydrates, amino acids, short-chain fatty acids, and other metabolites reflected the external environment, that is, the geographical location. In addition, the internal environment, namely the mode of delivery, also impacted the metabolite profile. Our findings support the hypothesis that human milk metabolites from healthy women vary across locations. Shifts on metabolites are associated to milk microbiota profiles suggesting a complex interlink between milk compounds. It is known that breast-feeding provides a personalized infant nutrition driving the infant gut development, immune system maturation and metabolic activities. Climate, lifestyle, environmental exposure, circadian rhythms, ethnic origin, population-specific variations and genetics would have an impact on metabolite profile explaining some of the variation observed in this study and request further research. Furthermore, our study highlights the potential interactions between human milk metabolites and microbes.

Therefore, we need to understand the pivotal relationship between environment-host-nutrition during pregnancy, lactation and early infancy due its impact for human health. This knowledge would enable the design personalized interventions to modulate milk bioactive compounds to be transferred through breastfeeding to the infants. Our data highlight the need for controlled- large-scale human studies across locations and the need to associated changes on milk bioactive compounds to infant growth, development, and health outcomes.

On this basis, we suggest the hypothesis that breast milk provides optimal immunological and metabolic guidance to the extrauterine world, although the differences reported between the locations and modes of delivery in terms of the metabolite composition might involve variations in infant development.

## Figures and Tables

**Table 1:** Human milk metabolites identified in breast milk samples and their probable origin. Chemical shifts in ppm are presented (in brackets).

Metabolite	Origin
*Amino acids and derivatives*	
**2-Hydroxybutyrate** (3.99)	endogenous
2-Hydroxyisovalerate (0.95)	endogenous
**Alanine** (1.47)	endogenous
Anserine (8.91)	diet
Creatinine (3.03)	endogenous
**Dimethylamine** (2.72)	endogenous
Glutamate (2.34)	endogenous
Glutamine (2.47)	endogenous
Carnitine (3.21)	endogenous
Histidine (7.09)	endogenous
Isoleucine (0.99)	diet
**Kynurenine** (6.81)	endogenous
**Leucine** (0.94)	diet
Methionine (2.62)	diet
**Methyl-histidine** (7.88)	endogenous
***N*-Acetyl lysine** (1.79)	endogenous
Phenylalanine (7.36)	endogenous
**Proline** (3.34)	endogenous
Taurine (3.25)	endogenous
Tryptophan (7.70)	diet
Tyrosine (3.06)	endogenous
**Urea** (5.77)	endogenous
Valine (0.98)	diet
*Energy metabolites*	
**3-hydroxybutyrate** (1.17)	endogenous
Citrate (2.69)	endogenous
**Creatine** (3.01)	endogenous
**Creatine-phosphate** (3.02)	endogenous
**Formate** (8.44)	endogenous
**Lactate** (1.32)	endogenous
NADH (8.46)	endogenous
*Neurotransmitters, growth factors and second messengers*	
4-Aminobutyrate (2.29)	endogenous
Putrescine (1.75)	microbial
**Spermidine** (2.61)	endogenous
**Spermine** (2.70)	endogenous
*Fatty acids and associated metabolites*	
4-Aminohippurate (2.29)	endogenous
**Acetate** (1.91)	endogenous
Acetylcholine (3.21)	endogenous
**Butyrate** (2.16)	microbial
Choline (4.06)	diet
**Ethanolamine** (3.13)	endogenous
**Glycero-3-phosphocholine** (3.22)	endogenous
LDL * (1.29)	endogenous
**Phosphocholine** (3.2)	endogenous
VLDL * (1.27)	endogenous
*Sugars and derivatives*	
1,6-anhydro-B-glucose (5.44)	diet
**2-fucosyllactose** (5.31)	endogenous
**3-fucosyllactose** (5.37)	endogenous
**Arabinose** (4.51)	microbial
**Fucosyl-α-1,3-*N*-acetylglucosamine** (5.14)	endogenous
**Fucosyl-α-1,4-*N*-acetylglucosamine** (5.01)	endogenous
Fucose (4.55)	endogenous
**Galactose** (4.57)	endogenous
Glucose (3.23)	endogenous
**Glucose-1-phosphate** (5.45)	endogenous
Lactose (3.75)	endogenous
**Lactodifucotetraose** (5.27)	endogenous
**Lacto-*N*-difucohexaose I** (5.18)	endogenous
**Lacto-*N*-difucohexaose II** (5.36)	endogenous
**Lacto-*N*-fucopentaose I** (5.31)	endogenous
**Lacto-*N*-fucopentaose III** (5.1)	endogenous
*Myo*-inositol (4.05)	endogenous
*N*-Acetylglucosamine (3.91)	endogenous
*Vitamins and nucleosides*	
**Riboflavin (B2)** (7.96)	diet
**Uridine** (5.92)	endogeonous
*Others*	
Ethanol (1.16)	diet
**Hippurate** (7.55)	microbial
**Propylene glycol** (1.14)	diet

Classification and probable origin inferred from “The Human Metabolome Database” [18]. Metabolites employed for statistical comparison are highlighted with bold letters. * LDL- and VLDL-like particles with similar NMR resonances, structure and mobility than plasma LDL and VLDL.

**Table 2:** Significant differences by country (*p*-value) in specific metabolites in human milk after vaginal or caesarean delivery.

Metabolite	China	Finland	Spain	South Africa
3-fucosyllactose	0.739	0.971	0.190	↑ 0.035 *
Alanine	0.853	0.739	0.280	↑ 0.022 *
Butyrate	0.579	0.579	0.436	↑ 0.006 *
Dimethylamine	0.971	0.035 *	↑ 0.035 *	0.002 *
Ethanolamine	0.631	0.579	↑ 0.000 *	0.661
Formate	↑ 0.035 *	0.684	0.796	0.006 *
Fucosyl-α-1,4-*N*-acetylglucosamine	0.631	0.035 *	0.247	0.002 *
Galactose	0.579	0.579	0.481	↑ 0.002 *
Hippurate	0.684	*0.063*	↑ 0.009 *	0.022 *
Kynurenine	0.143	*0.075*	↑ 0.011 *	0.028 *
Lactodifucotetraose	0.247	↑ 0.035 *	0.247	0.447
Leucine	0.631	↑ 0.043 *	↑ 0.009 *	1.000
Lacto-*N*-fucopentaose I and 2-fucosyllactose	0.353	1.000	0.971	0.035 *
Lacto-*N*-fucopentaose III	0.579	0.796	0.631	0.001 *
Methyl-histidine	0.353	0.684	↑ 0.023 *	0.043 *
Phosphocholine and acetylcholine	0.393	0.796	1.000	0.002 *
Proline	0.393	0.739	↑ 0.002 *	1.000
Propylene glycol	0.912	*0.089*	↑ 0.023 *	0.004 *
Riboflavin	0.529	0.353	↑ 0.002 *	0.400
Spermidine	0.579	0.353	↑ 0.000 *	0.604
Spermine	0.143	0.315	0.853	0.003 *
Urea	0.436	0.247	0.280	0.000 *
Uridine	0.529	0.912	0.019 *	0.243

* indicate *p*-values < 0.05. ***↑*** indicate significant higher levels in milk from caesarean section donors.

## Supplementary Materials

The following are available online at http://www.mdpi.com/2072-6643/10/10/1355/s1, Table S1: Age and pre-pregnancy body max index (BMI) of the participants in the study. Height was measured during the first visit at hospital and weight was self-reported, Table S2: Significantly (*p* < 0.05) spearman’s rank-order correlation between metabolites and maternal characteristics, Figure S1: Principal component analysis (PCA) scores plot (A) and loadings of the first principal component (B) and second principal component (C) scaling NMR data from all participant countries. Countries are indicated as red diamonds (China), green squares (Finland), navy blue triangles (Spain), pale blue triangles (South Africa), Figure S2: Principal component analysis (PCA) scores plot (A) and loadings of the first principal component (B) and second principal component (C) scaling NMR data from all participant countries without Spain. Countries are indicated as red diamonds (China), green squares (Finland), pale blue triangles (South Africa), Figure S3: Box and whisker plot showing statistical significant differences in metabolityes in breast milk samples after vaginal or caesarean delivery. LNFP III: lacto-N-fucopentaose III. Mann-Whitney U test significant level was considered at 0.05. Each bar represents the smallest observation, lower quartile (Q1), median, upper quartile (Q3) and largest observation. Circles and stars indicate outlier data.
