# Supplementary material for: Human Breast Milk NMR Metabolomic Profile across Specific Geographical Locations and Its Association with the Milk Microbiota
Source: Nutrients. 2018 Sep 21;10(10):1355. doi: 10.3390/nu10101355 (PMC6213536; doi:10.3390/nu10101355)
Supplement: Supplementary file 1 [file nutrients-10-01355-s001.pdf]

nutrients

MDPI

Supplementary Materials:

Table S1. Age and pre-pregnancy body max index (BMI) of the participants in the study. Height was measured during the first visit at hospital and weight was self-reported.

	Age (years)		Pre-Pregnancy BMI (kg/m²)	
	**Mean (Range)**	**SD**	**Mean (Range)**	**SD**
South Africa	34.05 (22–43)	6.3	25.75 (22.34–30.86)	2.3
Spain	33.35 (22–40)	4.1	24.30 (21.37–27.22)	1.4
China	32.25 (24–37)	3.6	21.71 (18.29–27.04) *	1.9
Finland	34.45 (23–42)	5.1	25.50 (20.57–31.86)	3.1
Total	33.53 (22–43)	4.9	24.31 (18.29–31.86)	2.8

* Indicate statistically significant differences in the same column among countries after one-way ANOVA at the level of $p < 0.05$.

Table S2. Significantly ($p < 0.05$) spearman's rank-order correlation between metabolites and maternal characteristics.

	Positive Correlation	Negative Correlation
Age	Glycero-3-phosphocholine, carnitine and lacto-N-fucopentaose III	
BMI	VLDL-like particles *, lactate, 2-Hydroxybutyrate, 2-fucosyllactose and lacto-N-fucopentaose I.	Phosphocholine and acetylcholine *, lacto-N-fucopentaose III, kynurenine, hippurate*, methyl-histidine* and riboflavin *

* Correlation significant at the level of $p < 0.01$.

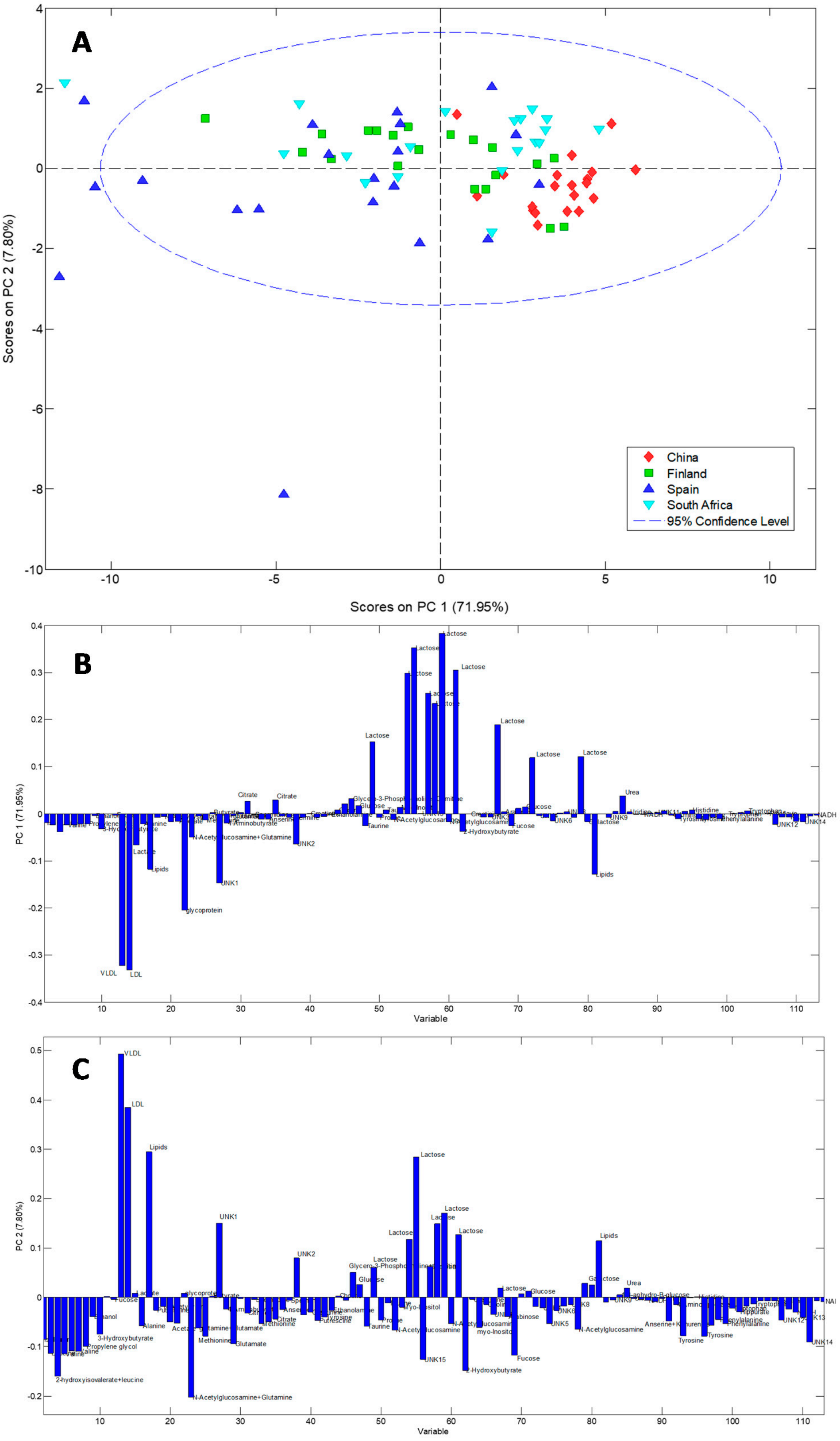

Figure S1. Principal component analysis (PCA) scores plot (**A**) and loadings of the first principal component (**B**) and second principal component (**C**) scaling NMR data from all participant countries. Countries are indicated as red diamonds (China), green squares (Finland), navy blue triangles (Spain), pale blue triangles (South Africa).

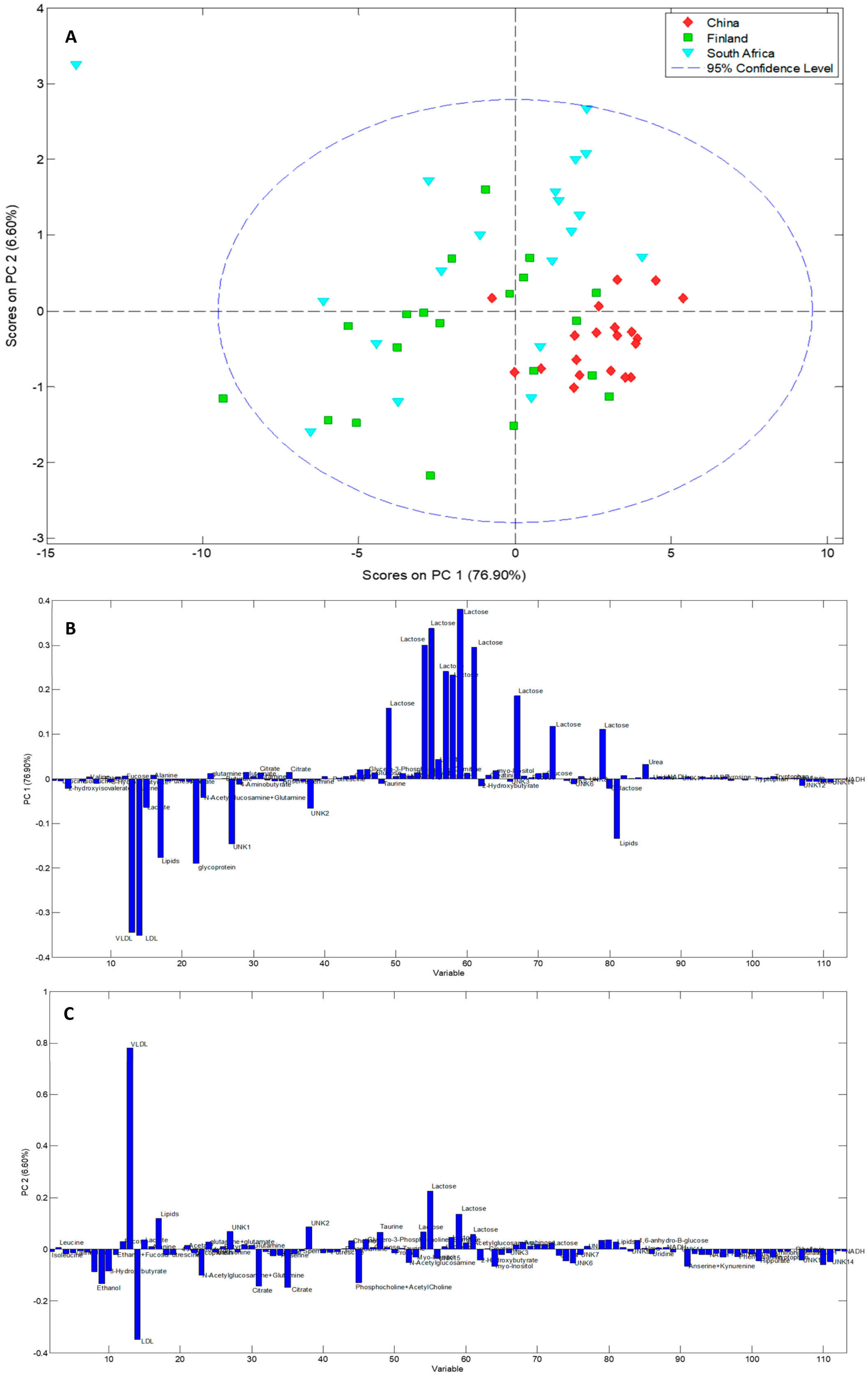

Figure S2. Principal component analysis (PCA) scores plot (**A**) and loadings of the first principal component (**B**) and second principal component (**C**) scaling NMR data from all participant countries without Spain. Countries are indicated as red diamonds (China), green squares (Finland), pale blue triangles (South Africa).

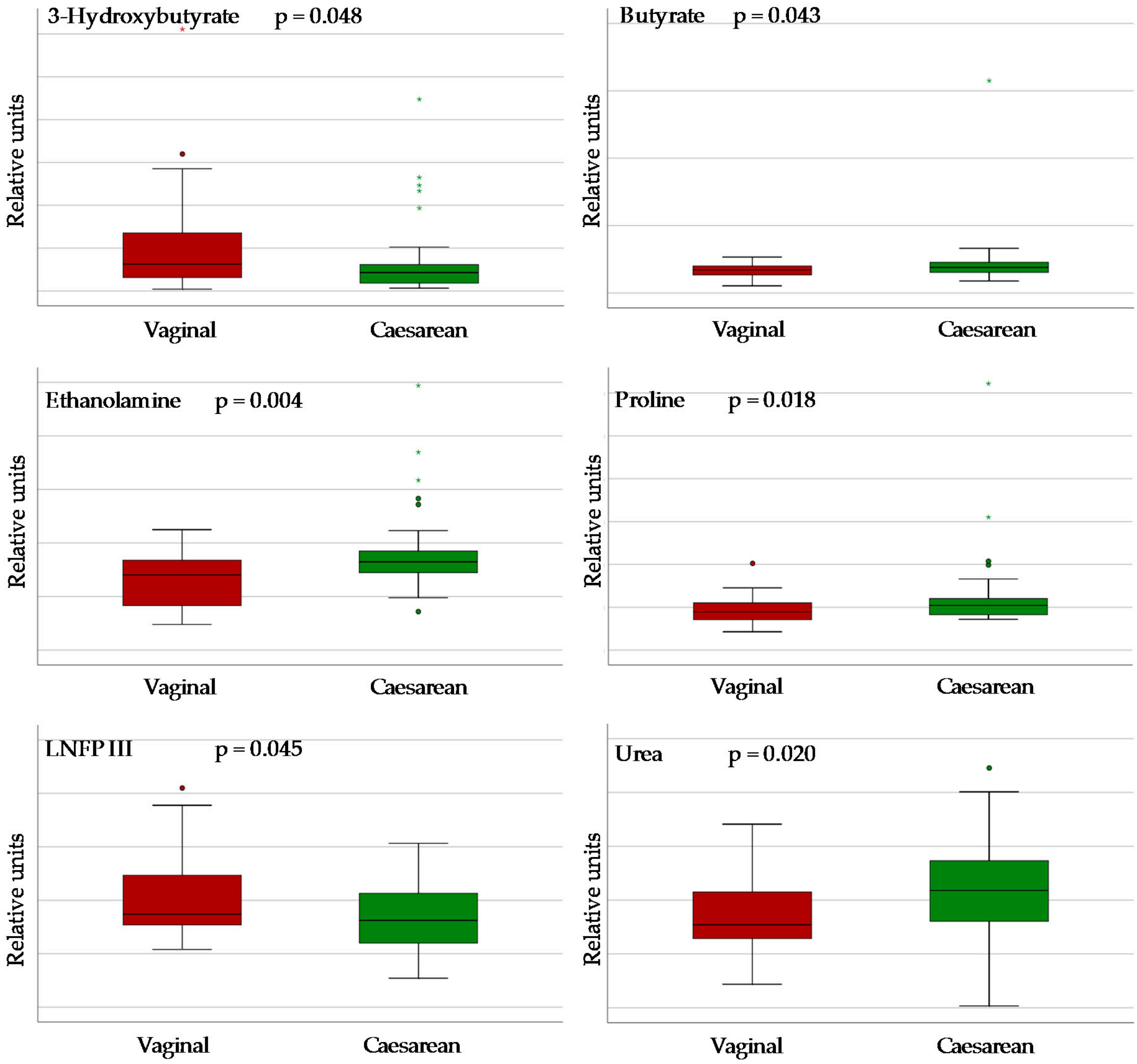

Figure S3. Box and whisker plot showing statistical significant differences in metabolityes in breast milk samples after vaginal or caesarean delivery. LNFP III: lacto-N-fucopentaose III. Mann-Whitney U test significant level was considered at 0.05. Each bar represents the smallest observation, lower quartile (Q1), median, upper quartile (Q3) and largest observation. Circles and stars indicate outlier data.
